# Effects of Slow Deep Breathing at High Altitude on Oxygen Saturation, Pulmonary and Systemic Hemodynamics

**DOI:** 10.1371/journal.pone.0049074

**Published:** 2012-11-12

**Authors:** Grzegorz Bilo, Miriam Revera, Maurizio Bussotti, Daniele Bonacina, Katarzyna Styczkiewicz, Gianluca Caldara, Alessia Giglio, Andrea Faini, Andrea Giuliano, Carolina Lombardi, Kalina Kawecka-Jaszcz, Giuseppe Mancia, Piergiuseppe Agostoni, Gianfranco Parati

**Affiliations:** 1 Department of Cardiology, San Luca Hospital, Istituto Auxologico Italiano, Milan, Italy; 2 Cardiologia Riabilitativa, Fondazione Salvatore Maugeri, IRCCS, Milan, Italy; 3 Department of Clinical Medicine and Prevention, University of Milano-Bicocca, Milan, Italy; 4 I Department of Cardiology and Hypertension, Jagiellonian University Medical College, Krakow, Poland; 5 Centro Cardiologico Monzino, IRCCS, and Department of Cardiovascular Sciences, University of Milan, Milan, Italy; Vanderbilt University Medical Center, United States of America

## Abstract

Slow deep breathing improves blood oxygenation (Sp_O2_) and affects hemodynamics in hypoxic patients. We investigated the ventilatory and hemodynamic effects of slow deep breathing in normal subjects at high altitude. We collected data in healthy lowlanders staying either at 4559 m for 2–3 days (Study A; N = 39) or at 5400 m for 12–16 days (Study B; N = 28). Study variables, including Sp_O2_ and systemic and pulmonary arterial pressure, were assessed before, during and after 15 minutes of breathing at 6 breaths/min. At the end of slow breathing, an increase in Sp_O2_ (Study A: from 80.2±7.7% to 89.5±8.2%; Study B: from 81.0±4.2% to 88.6±4.5; both p<0.001) and significant reductions in systemic and pulmonary arterial pressure occurred. This was associated with increased tidal volume and no changes in minute ventilation or pulmonary CO diffusion. Slow deep breathing improves ventilation efficiency for oxygen as shown by blood oxygenation increase, and it reduces systemic and pulmonary blood pressure at high altitude but does not change pulmonary gas diffusion.

## Introduction

From the point of view of oxygen gas exchange, human lungs are highly inefficient, as suggested by the 50–60 mmHg P_O2_ gap between atmosphere and arterial blood observed at sea level. Indeed, some animal species can reach much higher altitudes than humans without supplement O_2_ due to several reasons including a lower P_O2_ gap between atmosphere and arterial blood [1]. Moreover, in humans hypoxia is often observed in diseases such as lung and cardiac disease or in specific conditions such as high altitude (HA). Indeed, an acute exposure to HA hypoxia, as during hiking or climbing, is associated with reduced P_O2_ in alveolar air and in arterial blood. In turn, hypoxemia activates a chemoreflex response leading to increased ventilation, which results in hypocapnia and respiratory alkalosis. Exposure to HA is also associated with pulmonary hypertension and lung fluid accumulation, both of which further contribute to hypoxemia and, in some cases, lead to high altitude pulmonary edema (HAPE) [2], [3].

Efficiency of ventilation for oxygen may be improved by changing the respiratory pattern in order to optimize the partitioning between alveolar ventilation and airway ventilation, being that the latter useless in terms of gas exchange. This has been reported by Yoga practice [4] or by regular breathing as obtained during regular rosary praying [5]. Controlled breathing with low rate and high tidal volume, the so called “slow deep breathing”, has also been shown to improve the efficiency of ventilation by increasing alveolar and reducing dead space ventilation [6]. Slow deep breathing may also improve arterial oxygenation by increasing alveolar volume and gas exchange at the alveolar capillary membrane level. The latter particularly increases when interstitial lung fluids are increased. Indeed, it has been reported that paced slow deep breathing improves blood oxygenation in subjects chronically exposed to HA [7] and in patients with congestive heart failure or with chronic pulmonary obstructive disease [6], [8], [9], [10]. Slow deep breathing might also counteract some hemodynamic effects of hypobaric hypoxia at HA, including the increase in systemic blood pressure [11], given the evidence that device-guided slow deep breathing reduces elevated blood pressure in hypertensive patients [12], [13], [14].

Thus, aim of the present study was to assess whether slow deep breathing improves ventilation efficiency in healthy subjects at HA. Specifically, we evaluated if slow deep breathing increases oxygen saturation during acute and prolonged exposure to HA and the mechanisms behind it, being the former but not the latter associated to an increase of extravascular lung fluids [15], [16].

## Methods

The present paper reports the data obtained in two groups of subjects and under different hypoxic conditions, i.e. during acute (Study A) and prolonged (Study B) exposure to HA, within the frame of the HIGH altitude CArdiovascular REsearch (HIGHCARE) project. Studies are registered as EudraCT Number [2010-019986-27] (Study A) and EudraCT [2008-000540-14] (Study B).

### Design and Setting

Study A was performed at Capanna Regina Margherita (CRM, 4559 m, Monte Rosa, the Alps). The ascent to CRM from sea level (Milan, 140 m) was divided in two days including an overnight stay at 3672 m. Subjects were brought by car and cable car up to 3200 m, and the remaining part of the ascent was covered by hiking. Overall, the ascent from sea level to CRM took about 24 hours. Study tests were performed on the second and on the third full day of permanence at CRM.

In Study B, participants travelled by air from sea level (Milan, 140 m) to Kathmandu (Nepal, 1355 m) where they stayed for three days. Then they were brought again by air transport to 3400 m (Namche Bazaar), where they stayed for another three days. After that, a 5-day trekking led them to Mt. Everest South Base Camp (5400 m) where they remained for 12 days. Study tests were performed on 5^th^ –9^th^ day of permanence at 5400 m (i.e. after a total of 12–16 days of high altitude exposure).

In both studies, each subject underwent: 1) baseline assessment after at least 15 minutes of rest at a spontaneous breathing rate, 2) 15 minutes of slow deep breathing exercise at a rate of 0.1 Hz (6 breaths per minute) paced by a digital metronome (RESPeRATE, Intercure Ltd, Lod, Israel), and 3) recovery (5 minutes – Study A, 30 minutes – Study B) at a spontaneous breathing rate. The study sessions were not preceded by food intake or strenuous exercise, and data collection was performed in sitting position and in conditions of controlled ambient temperature.

The RESPeRATE device used for breath pacing includes a battery-operated hand-held computerized box attached to headphones and to a belt-type respiration sensor. Breathing is guided by a 2-tone melody heard by the subject, who is asked to inhale when a high tone is heard and exhale when a low tone is heard. Breathing rate reduction is mainly obtained by prolonging the expiration phase so that the ratio between inspiration and expiration duration is about 1∶2. In its normal operation mode, the device provides a gradual reduction of breathing rate; however, for the purposes of this study, it was used in a fixed frequency mode at 6 breaths per minute. Before starting the study procedures, the subjects were briefly acquainted with the device. The appropriate performance of the slow breathing was constantly monitored.

### Subjects

We enrolled healthy volunteers of both sexes, living permanently at less than 500 m. Subjects were sought among the personnel of the organizing institute and through personal contacts and identified based on their availability for the high-altitude expedition, on their willingness to undergo the testing, and on the physical ability to perform the ascent to high altitude location. Exclusion criteria were: known cardiovascular disease, repeated exposures to altitudes >3000 m in the 8 months preceding the expedition, history of severe mountain sickness, and pregnancy. Professional athletes or climbers were also not included in the study.

The study protocols were approved by the Ethics Committee of Istituto Auxologico Italiano. All subjects gave their written informed consent to participate in the study. During the ascent to high altitude and during the subsequent descent, subjects were assisted by professional Alpine climbers and guides.

### Measurements

**Figure 1:**
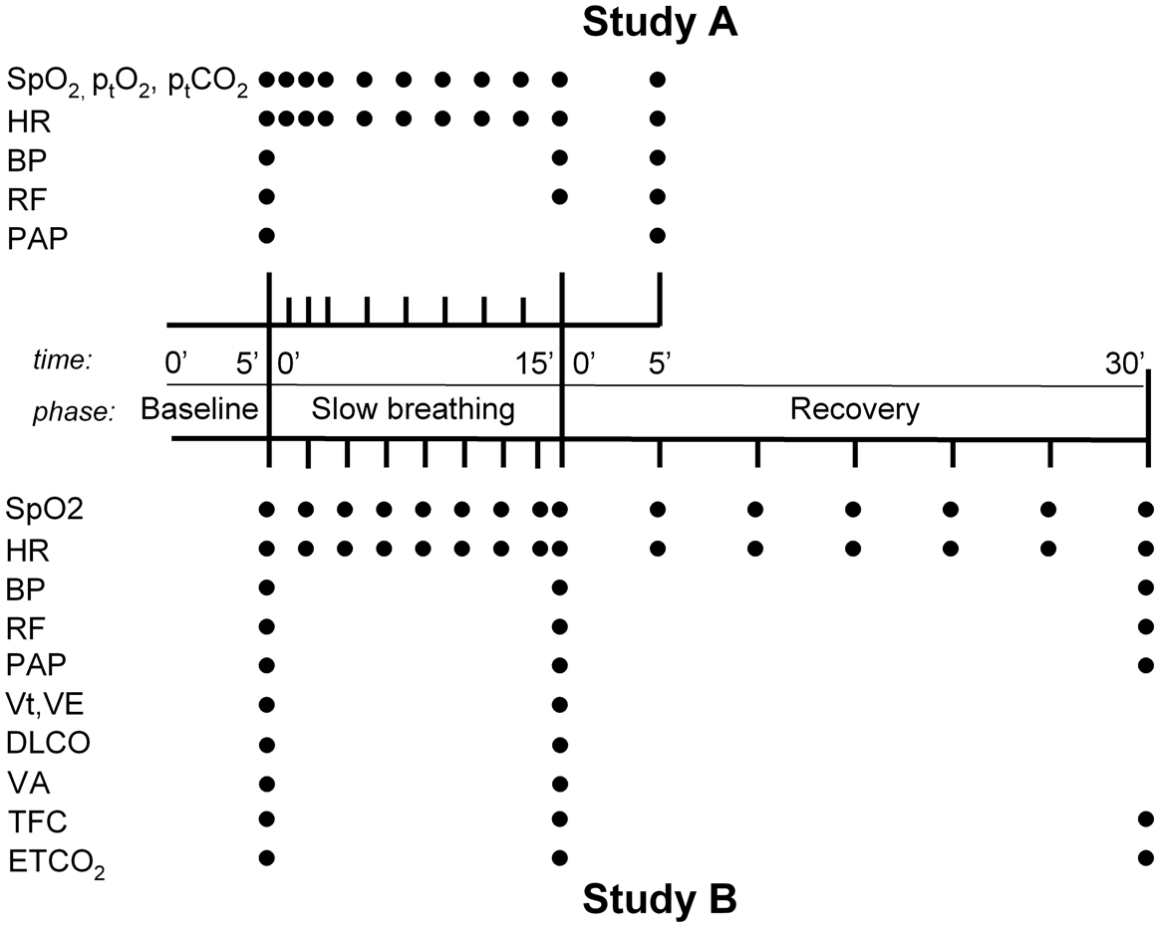
Schematic representation of the sequence of data collection in studies A and B. Sp_O2_, blood oxygen saturation; Pt_O2_, transcutaneous oxygen partial pressure; Pt_CO2_, transcutaneous CO_2_ partial pressure; HR, heart rate; BP, blood pressure; RF, respiratory frequency; PAP, pulmonary artery pressure; Vt, tidal volume; VE, minute ventilation; Dl_CO_, pulmonary CO diffusion; VA = alveolar volume; TFC, thoracic fluid content; Pet_CO2_, end tidal CO_2_ pressure in the exhaled air.

The primary variable of interest in this study was blood oxygen saturation (Sp_O2_), while the secondary variables included arterial blood pressure (BP), heart rate (HR), respiratory frequency (RF) and systolic pulmonary artery pressure (PAP). Additional parameters that were assessed included transcutaneous measurement of P_O2_ (Pt_O2_) and of P_CO2_ (Pt_CO2_) in Study A, and end tidal CO_2_ level in the exhaled air (Pet_CO2_), thoracic fluid content (TFC) by impedance cardiography, tidal volume (Vt), minute ventilation (VE), alveolar volume (VA), and pulmonary CO diffusion (Dl_CO_) in Study B. The timing of measurements in both studies is reported in Figure 1.

Sp_O2_ was measured by pulse oximetry (Life Scope I, Nihon Kohden, Tokyo, Japan (Study A) and Tuffsat, GE Healthcare, Milwaukee, WI, USA (Study B)). Resting BP was measured in sitting position according to European Society of Hypertension guidelines (O’Brien et al., 2003) with a validated oscillometric device (OMRON M5-I, Omron, Tokyo, Japan in Study A and Microlife A100Plus Microlife, Windau, Switzerland in Study B), and HR was obtained from a pulse oximeter. Pulse pressure (PP) was calculated as systolic (S) minus diastolic (D) BP. Echocardiography was performed with a Vivid I device (GE Medical Systems, Milwaukee, WI, USA), and pulmonary artery pressure (PAP) was calculated by the analysis of tricuspid regurgitation provided by the continuous Doppler signal (assuming a right atrial pressure of 5 mmHg). Transcutaneous P_O2_ and P_CO2_ were continuously measured with TCM4 device (Radiometer, Copenhagen, Denmark) after obtaining stable values with the probe placed on the forearm. Impedance cardiography was performed for 5 minutes in supine position with NICCOMO Cardioscreen (Medis GmbH, Ilmenau, Germany). RF was assessed by counting breaths over 1 minute. Vt, VE, VA and Dl_CO_ were assessed with Sensor Medics 2200 device (Yorba Linda, CA, USA). VA and Dl_CO_ were measured with the single breath – constant expiratory flow technique and calculated according to current guidelines (American Thoracic Society, 1995). Tracer gas for VA measurements was CH4. Dl_CO_ was corrected for hemoglobin and then for differences in inspired O_2_ (Pi_O2_), due to differences in barometric pressure between sea level and altitude, and concentration according to the formulas: Dl_CO_ measured x (10.15+ haemoglobin)/1.7×haemoglobin [17] and: Dl_CO_ measured Hb corrected x (1.0+0.0031(Pi_O2_–150)) [18], respectively. Pet_CO2_ was measured with Microcap Plus capnograph (Oridion Capnography Inc., Needham, MA, USA).

### Sample Size

Both studies were performed within the frame of larger research projects, and therefore the overall sample size was not determined based on the requirements of slow breathing substudies. However, assuming a SD of Sp_O2_ change of 7%, 19 subjects were needed in order to detect a 5% Sp_O2_ change with 90% power and alpha = 0.05. This number was exceeded in both studies.

### Data Analysis

Statistica 8.0 (StatSoft, Tulsa, USA) software was used for statistical analysis. Means ± standard deviations (SD) were used for descriptive statistics of continuous variables. Repeated measures analysis of variance was used to assess the changes in the study variables in different phases of the protocol. Bonferroni correction was used for post-hoc comparisons, except for comparisons of Sp_O2_, Pt_O2_ and Pt_CO2_ values obtained at multiple time points during slow breathing and recovery, where, because of the large number of comparisons involved, two-sided Dunnet test was performed, taking the corresponding baseline values as the reference. Pearson’s correlation coefficients were computed to identify the determinants of the size of Sp_O2_ change during slow breathing versus baseline and their relative significance was assessed in multiple linear regression models. Pearson’s chi-square test was used to compare gender composition between subgroups. Throughout the study, a p<0.05 was taken as the level of statistical significance.

## Results

Thirty male and nine female subjects (77% and 23%) aged 24–61 years (mean 38.8±11.0) with a mean body mass index (BMI) of 22.9±2.8 kg/m^2^ were included in study A. The population of Study B (n = 28) was very similar to that of Study A (age 38.9±10.6, BMI 22.9±2.8) except for a higher proportion of female subjects [16 (57%) males and 12 (43%) females].

### Changes in Ventilatory Variables

At sea level, all subjects were normotensive and their Sp_O2_ was invariably >95%. At HA, all subjects achieved the required respiratory frequency during device-guided slow breathing exercise.

**Figure 2:**
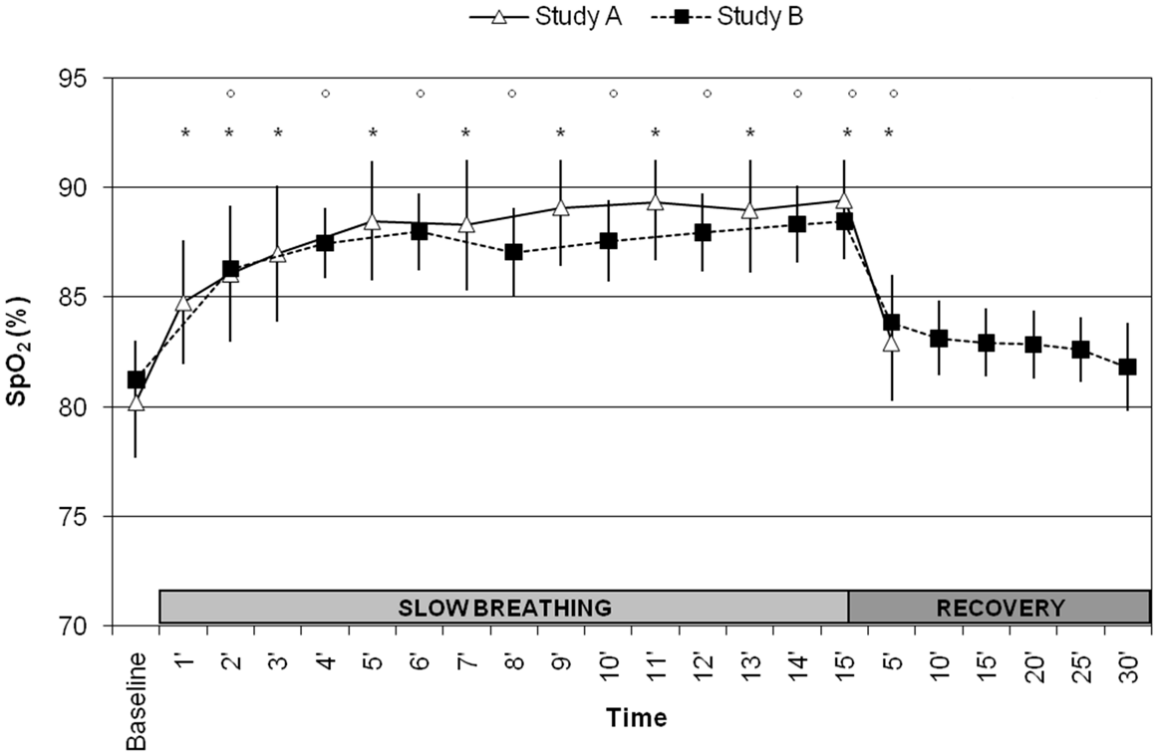
Changes in Sp_O2_ (upper panel), Pt_O2_ (middle panel) and Pt_CO2_ (lower panel) during slow breathing exercise and recovery. Differences vs. baseline with p<0.05 are identified with * (Study A) or ° (Study B) symbols. Vertical bars denote 95% CI of the mean.

In both studies, slow breathing exercise at HA was associated with a significant increase in Sp_O2_ from baseline value (from 80.2±7.7% to 89.5±8.2% in Study A and from 81.0±4.2% to 88.6±4.5 in Study B, both p<0.001) (Figure 2). A significant increase in Pt_O2_ and a significant reduction in Pt_CO2_ occurred during slow deep breathing (Study A, Table 1), together with a proportional increase in tidal volume and, consequently, with no change in minute ventilation (Study B). No significant changes were induced by slow deep breathing in VA and in Dl_CO_ (Study B). During the recovery phase, all variables returned to baseline values, with the exception of blood oxygenation (Sp_O2_ and Pt_O2_), which remained somewhat higher after 5 minutes of recovery compared with baseline (Table 1, Figure 2).

**Table 1:** Study variables assessed at high altitude in baseline condition, after 15 minutes of slow breathing exercise and after 5 and 30 minutes of recovery.

Variable	Study	N	Baseline	Slow breathing (15′)	Recovery		p (overall)
					5′	30′	
Sp_O2_ (%)	A	39	80.2±7.7	89.5±8.2 ***	82.9±8.1 *†††	–	<0.001
	B	28	81.0±4.2	88.6±4.5 ***	83.8±5.8 *†††	81.7±5.4 †††	<0.001
P_tO2_ (mmHg)	A	37	42.8±7.6	56.9±11.4 ***	47.6±11.3 **†††	–	<0.001
	B	–	–	–	–	–	–
P_tCO2_ (mmHg)	A	39	26.4±4.0	22.5±5.9 ***	25.8±4.8 †††	–	<0.001
	B	–	–	–	–	–	–
Et_CO2_ (mmHg)	A	–	–	–	–	–	–
	B	28	21.4±3.9	20.1±4.5	–	21.2±3.6	0.15
SBP (mmHg)	A	39	126.0±14.5	118.5±16.0 ***	122.7±16.6 *††	–	<0.001
	B	28	125.2±15.4	115.0±14.5 ***	–	127.9±17.0 †††	<0.001
DBP (mmHg)	A	39	75.0±15.2	68.7±14.1 ***	69.8±15.1 ***	–	<0.001
	B	28	79.4±9.5	78.3±9.5	–	83.5±9.2 *††	<0.001
PP (mmHg)	A	39	51.0±13.8	49.8±13.4	52.9±12.6 †	–	0.052
	B	28	45.8±11.4	36.7±10.6 ***	–	44.4±13.1 †††	<0.001
HR (bpm)	A	39	81.6±11.4	79.3±11.3	81.2±12.0	–	0.27
	B	28	87.6±14.8	83.1±12.3	84.5±13.3	84.1±16.8	0.09
sPAP (mmHg)	A	9	33.7±4.5	–	28.7±5.0 **	–	0.008
	B	28	33.1±10.9	29.2±10.3 ***	–	31.5±10.4	0.001
RF (1/min)	A	39	17.6±5.3	6.0±0.1 ***	17.3±6.2 †††	–	<0.001
	B	28	16.0±3.8	6.0±0.2 ***	–	14.1±4.7 †††	<0.001
Vt (l)	A	–	–	–	–	–	–
	B	18	1.5±0.6	3.0±0.7 ***	–	–	<0.001
VE (l)	A	–	–	–	–	–	–
	B	17	16.9±6.8	17.6±3.8	–	–	0.66
Dl_CO_ (ml/min/mmHg)	A	–	–	–	–	–	–
	B	17	30.0±6.6	34.7±7.1	–	–	0.27
VA (l)	A	–	–	–	–	–	–
	B	17	6.65±0.85	6.71±0.91			0.59
TFC (ml/kg)	A	–	–	–	–	–	–
	B	24	36.1±6.4	36.0±6.0	–	34.9±6.8	0.25

Data are separately shown for Study A and B.

* -p<0.05, **-p<0.01, ***, p<0.001 vs. baseline; † - p<0.05, †† - p<0.01, †††, p<0.001 vs. slow breathing.

Sp_O2_, blood oxygen saturation; p_tO2_– transcutaneous oxygen partial pressure; pt_CO2_– transcutaneous CO_2_ partial pressure; Et_CO2_– end tidal CO_2_ pressure in the exhaled air; SBP – systolic blood pressure; DBP – diastolic blood pressure; PP – pulse pressure; HR – heart rate; sPAP – systolic pulmonary artery pressure; RF – respiratory frequency; Vt – tidal volume; VE – minute ventilation; VA – alveolar volume; Dl_CO_ - pulmonary CO diffusion; TFC – thoracic fluid content.

### Changes in Hemodynamic Variables

A significant reduction in SBP was found in both studies at the end of the slow breathing session, with SBP values which remained lower than at baseline after 5 minutes (Study A) but not after 30 minutes (Study B) of recovery. The corresponding changes in DBP and PP are shown in Table 1. HR tended to decrease during slow deep breathing, but the changes were not significant. Pulmonary artery pressure was significantly reduced at the end of slow deep breathing exercise (Study B) and after 5 minutes of recovery (Study A). There were no significant changes in thoracic fluid content as quantified by impedance cardiography in Study B (Table 1).

### Predictors of Response to Slow Breathing Exercise

In Study A and B, the only variable which significantly predicted the increase of Sp_O2_ induced by slow deep breathing was the Sp_O2_ baseline value itself. This relationship was inverse in nature (Study A: r = −0.38, p = 0.017, Study B: r = −0.48, p = 0.010), i.e. a lower baseline Sp_O2_ predicted a larger increase in Sp_O2_ with the slow breathing exercise.

## Discussion

Our paper offers for the first time information on the respiratory and hemodynamic effects of device-guided slow deep breathing in healthy lowlanders exposed to HA. Our main result is that in healthy subjects exposed to HA, i.e. to a low ambient-air P_O2_, the change in breathing pattern from a spontaneous rate to a paced frequency of 6 breaths per minute was associated with an improvement of ventilation efficiency, as shown by the significant increase in blood oxygen saturation. This was the case both for acute (Study A) and prolonged (Study B) exposure to HA hypoxia. This increase occurred rapidly and was maintained throughout the slow deep breathing period. Most of the improvement of blood oxygenation was lost within 5 minutes after restoration of spontaneous breathing pattern, and no differences compared with baseline were evident after 30 minutes. Our study extends the previous reports on the benefits of slow deep breathing intervention in other hypoxic conditions, such as in patients with chronic pulmonary obstructive disease [10], in hypoxic patients with heart failure [6], and in subjects living permanently at HA [7]. In all these studies, the observed changes were less pronounced than in our paper, a discrepancy which is explained by the higher baseline Sp_O2_ values they reported (about 90%) as compared to the conditions of our studies (about 80%).

In the present study, we showed for the first time the time course of the response to slow deep breathing, showing that the maximum effect is reached after about 5 minutes and is subsequently maintained. Moreover, we reported for the first time data on the recovery period. In Study B, we extended the recovery period to 30 minutes, which allowed us to observe a progressive reduction of slow deep breathing effects, which are at their highest after 5 minutes, but some continue up to 30 minutes after its termination.

Baseline Sp_O2_ was similar in both studies despite the difference in altitudes of about 900 m. This may be explained by the fact that, at the same altitude, locations closer to the equator (like Nepal) are characterized by a somewhat higher atmospheric pressure, compared with locations at higher latitudes (like the Alps). Another possible explanation is the longer acclimatization time in Study B, associated with lower lung fluid content than in Study A. Indeed, prolonged residence at HA is associated with a progressive reduction in the initially increased interstitial lung liquid content, and with an improvement in alveolo-capillary gas exchange [15], [16]. Interestingly, slow deep breathing induced similar Sp_O2_ changes both under acute and prolonged exposure to HA regardless of lung fluid content (Figure 2). Thus, the effects of slow deep breathing on Sp_O2_ are likely to be independent from lung fluid content.

Our study provides some information on the possible mechanisms responsible for Sp_O2_ increase during slow deep breathing. In particular, ventilatory variables assessed in Study B indicate that this increase is not due to changes in minute ventilation during slow deep breathing, and indeed, the reduced respiratory rate is compensated by a proportionally increased tidal volume, but total ventilation is the same. However, the reduction of Pt_CO2_ during slow deep breathing exercise in Study A and the Sp_O2_ increase in both studies suggest that slow deep breathing improves the efficiency of ventilation. The lack of reduction of Pet_CO2_ in Study B (table 1) is not in contrast with this interpretation of our findings but merely a technical consequence of the measurement technique. Indeed, Pet_CO2_ pressure, due to the shape of the CO_2_ curve during expiration, is higher with lower respiratory frequency. Therefore, a reduction in Pa_CO2_ may actually have occurred during slow deep breathing in both studies. However, several other mechanisms may be hypothesized to participate in explaining our findings. First of all, the increased tidal volume might have mechanically modified the characteristics of the alveolar wall, thus facilitating gas exchange in a condition such as HA, where some degree of interstitial pulmonary edema is likely [3], [19]. Indeed, deep tidal volumes may lead to increase of fluid clearance by lymphatics [20] and to increase of venous return and, therefore, cardiac output. However, the latter does not seem to be a major component at least under prolonged exposure to HA (Study B), since slow deep breathing induced no significant change in lung CO diffusion (Dl_CO_), which is cardiac-output dependent, nor in thoracic fluid content as assessed by impedance cardiography. Besides, the increase in tidal volume [21] occurring with slow deep breathing may lead to alveolar recruitment and thus to a net increase in the surface available for gas exchange. However, anatomical VA was unchanged, but we measured with VA the total VA which is not the alveolar space actually used during spontaneous or paced breathing. We are likely to have increased the used alveolar volume with slow deep breathing and, consequently, we have reduced dead space minute ventilation and the dead space to tidal volume ratio to a percentage, as previously reported for heart failure patients during slow deep breathing exercise [7]. Furthermore, the transcutaneous P_O2_ and P_CO2_ values (table 1), if used as surrogates for arterial values in the conventional alveolar gas equation, show a reduction in the Alveolar-arterial P_O2_ difference consistent with improvements in ventilation-perfusion mismatch. In addition, pulmonary arterial pressure was reduced by slow deep breathing in both studies, as in congestive heart failure patients, suggesting pulmonary perfusion improvement [9]. Moreover, because slow deep breathing is associated to a reduction of sympathetic tone (see below), the improvement of ventilation/perfusion matching may also originate by more respiratory sinus arrhythmia [22]. Finally, the reduction of sympathetic tone could lead to a reduction in metabolic rate, which, possibly combined with an increase of cardiac output, may lead to an increase of mixed venous P_O2_ and thus less admixture. All together, our data suggest that the benefits from slow deep breathing exercise are due to an improvement in ventilation mechanics, in pulmonary perfusion and in ventilation/perfusion matching, and possibly to a reduction of the metabolic rate.

Another interesting result of our study is that slow deep breathing affected not only pulmonary, but also systemic hemodynamics. As shown in a recent paper by our group [11], exposure to HA is associated with an increase in arterial blood pressure over 24 hours, likely due to the elevated sympathetic drive in this condition, related to the chemoreflex response to hypoxia. Slow deep breathing induced a reduction in blood pressure (mainly in SBP) at HA, which partly persisted early in the recovery phase (Study A) and was no longer present after 30 minutes of recovery (Study B). This acute blood pressure lowering effect of slow deep breathing may be related to the ability of this manoeuvre to increase baroreflex and reduce chemoreflex sensitivity [8], [23], resulting in a sympathetic inhibitory action, as recently directly shown by Oneda et al. [24]. The blood pressure reduction observed in our study is in line with data obtained in previous studies that proposed regular and repeated performance of slow deep breathing exercise at sea level as a nonpharmacological approach to the treatment of hypertension [12], [13], [14]. These studies have also emphasized that this effect may originate from an enhanced sensitivity of the baroreflex and/or a reduced sensitivity of the chemoreflex [4], [23].

Our study has a few limitations, which need to be acknowledged. Firstly, based on previous studies [13], [14], [23], [25], the duration of the slow deep breathing manoeuvre was 15 minutes, and therefore the effects of a more prolonged or repeated slow deep breathing at HA remain unexplored. Secondly, none of our subjects had HAPE, and therefore we were not able to directly test the efficacy of slow deep breathing in such condition. Thirdly, due to logistic limitations, we were not able to directly assess alveolar ventilation and dead space ventilation, but such information can be extrapolated from the present study data and from a previous report in hypoxic patients with heart failure [6].

In conclusion, slow deep breathing induced a significant improvement in ventilation efficiency as shown by Sp_O2_ increase in healthy subjects exposed to HA. This improvement was most likely due to a reduction of dead space ventilation and an increase in alveolar ventilation, and was associated to a reduction of both pulmonary and systemic BP levels, both elevated at HA. This intervention is easy and cheap. The usefulness of slow deep breathing should however be tested in large scale studies on hypoxemic patients and at HA in subjects with HAPE.
